# A Liver-X-Receptor Ligand, T0901317, Attenuates IgE Production and Airway Remodeling in Chronic Asthma Model of Mice

**DOI:** 10.1371/journal.pone.0092668

**Published:** 2014-03-28

**Authors:** Ying Shi, Xiantao Xu, Yan Tan, Shan Mao, Surong Fang, Wei Gu

**Affiliations:** Department of Respiratory Medicine, Nanjing First Hospital, Nanjing Medical University, Nanjing, China; Clermont Université, France

## Abstract

The liver-X-receptors have shown anti-inflammatory ability in several animal models of respiratory disease. Our purpose is to investigate the effect of LXR ligand in allergen-induced airway remodeling in mice. Ovalbumin-sensitized mice were chronically challenged with aerosolized ovalbumin for 8 weeks. Some mice were administered a LXR agonist, T0901317 (12.5, 25, 50 mg/kg bodyweight) before challenge. Then mice were evaluated for airway inflammation, airway hyperresponsiveness and airway remodeling. T0901317 failed to attenuate the inflammatory cells and Th2 cytokines in bronchoalveolar lavage fluid. But the application of T0901317 reduced the thickness of airway smooth muscle and the collagen deposition. Meanwhile, T0901317 treatment evidently abolished the high level of OVA-specific IgE, TGF-β1 and MMP-9 in lung. So LXRs may attenuate the progressing of airway remodeling, providing a potential treatment of asthma.

## Introduction

Liver X receptors (LXRα/NR1H3 and LXRβ/NR1H2) are members of the nuclear receptor family that play central roles in cholesterol metabolism through controlling the transcription of genes such as ATP-binding cassette A1 (*ABCA1*) [1], [2], [3]. Activation of LXR inhibits the expression of several proinflammatory genes such as *iNOS* and *COX*2, *IL-*6 and *IL-*1, the chemokines monocyte chemoattractant protein-1 (*MCP*-1) and matrix metallopeptidase 9 (*MMP*-9) [4], [5]. LXRα is highly expressed in the liver and at lower levels in lung, glands, intestine and kidney, whereas LXRβ is ubiquitously expressed.

Bronchial asthma is an allergic disease manifests as symptoms such as wheezing, breathlessness, chest tightness and coughing. Cardinal features of the disease are airway inflammation, airway hyperresponsiveness (AHR) and airway remodeling [6]. Administration of LXR ligand showed inhibitory roles in lung inflammatory responses [7], [8], [9], [10] and anti-proliferative effects on smooth muscle [11], [12]. LXR also showed therapeutic roles in murine model of allergic contact dermatitis [13]. These suggest that LXRs are potential targets in asthma prevention and treatment.

As we all know, asthma is a multifactorial and complex disease in which the repetition of allergen challenges leads to airway remodeling eventually. The structural changes of the airway wall include epithelial denudation, subepithelial fibrosis, mucus gland hypertrophy, myofibroblast and smooth muscle proliferation, and angiogenesis (14, 15). In patients with severe asthma, airway remodeling is associated with the airway thickening, airway flow limitation and AHR (6). Here we investigated the potential ability of LXRs on airway remodeling in murine models of asthma.

## Methods

### Mice and ethics statement

Eight week old female BALB/c mice (each weighing approximately 20 g) were purchased from Shanghai Laboratory Animal Inc. The mice were allowed sterilized tap water and standard rodent chow in a specific pathogen-free biosafety level 3 facility. The animal experiment was approved by Nanjing Medical University according to the guidelines of the Institutional Animal Care and Use Committee (Permit Number: 20110217). Animal experiments were carried out in strict accordance with the regulations in the Guidance Suggestions for the Care and Use of Laboratory Animals issued by the Ministry of Science and Technology of the People's Republic of China. All surgery was performed under anesthesia and all efforts were made to minimize suffering.

### Antigen sensitization, challenge and LXRs ligand treatment

T0901317 (Sigma-Aldrich, USA) was dissolved in dimethylsulfoxide (DMSO) to make a concentration of 100 mg/ml as a stock solution.

Mice were randomly divided into four groups, namely control group, ovalbumin (OVA) group, dexamethasone (DEX) group and T0901317 group. On days 0, 7 and 14, mice were immunized by intraperitoneal injection of 100 μg chicken egg OVA (Grade V, Sigma, USA) adsorbed to 100 μL adjuvant (Inject Alum; Pierce, Rockford). From days 21, the OVA group, the mice of DEX group and T0901317 group were exposed to an aerosol of 1% OVA for 30 minutes/day, 3 days/week for 8 weeks. Mice of DEX group received intraperitoneal injections of DEX (1 mg/kg) 2 hours before every OVA challenge. Mice of T0901317 group received T0901317 (12.5, 25, 50 mg/kg bodyweight) orally 2 hours before OVA challenge. In the dose range, T0901317 has shown the inhibitory ability to inflammation [9], [16]. Mice of the control group were sham challenged with PBS.

There were two protocols employed to investigate the effect of T0901317 in this modeling system. Part of the first one was designed to demonstrate that T0901317 actually activated the LXR in this modeling system by measuring a marker of this receptor activation, ABCA1 mRNA expression. Additionally, we wanted to determine the impact of the agonist on OVA-induced inflammation and remodeling.

### Quantitative mRNA analysis of ABCA1

24 hours after the first challenge, some mice were anesthetized using an intraperitoneal injection of ketamine (100 mg/kg) and xylazine (10 mg/kg). Then mice were culled by cervical dislocation under anesthesia. Left lungs were collected and stored at −80°C. ABCA1 gene expression was assessed using real-time TaqMan RT-PCR. Total lung RNA was extracted with TRIzol Reagent (invitrogen) according to the manufacturer’s instructions. The purity and concentration of RNA were determined on a spectrophotometer. First-strand cDNA was synthesized from equal amounts of RNA with a reverse transcriptase reagent kit (TakaRa Biotechnology, Inc). mRNA levels were determined by real-time PCR analysis with SYBR Premix Ex Taq (TakaRa Biotechnology, Inc). Primer sequences employed for ABCA1 were as follows: sense 5′-AGTGATAATCAAAGTCAAAGGCACAC-3′, antisense 5′-AGCAACTTGGCACTAGTAACTCTG-3′; for β-actin, sense 5′-TAAAGACCTCTA TGCCAACACAGT-3′, antisense 5′-CACGATGGAG GGGCCGGACTCTTC-3′. PCR reactions were performed in an ABI Prism 7300 Sequence Detection System using the recommended reaction conditions: 95°C for 10s, then 40 cycles of 95°C for 5 s and 60°C for 31 s. To control for the specificity of the amplification products, a melting curve analysis was performed. The gene specific threshold cycle (Ct) for each sample was corrected by subtracting the Ct for the housekeeping gene β-actin (ΔCt). PBS treated controls were chosen as the reference samples, and theΔCt for all experimental samples were subtracted by the ΔCt for the control samples (ΔΔCt). The magnitude change of test gene mRNA was expressed as 2^−ΔΔCt^.

### Airway physiology

Airway responsiveness was measured 24 h after the last OVA challenge using an AniRes 2005 Lung Function System (Bestlab, Beijing, China). The mice were anesthetized with numbutal (60 mg/kg I.p.). The trachea was then surgically exposed and connected with a computer-controlled ventilator via an intratracheal tube. The respiratory rate and the time ratio of expiration/inspiration were pre-set at 90/min and 1.5:1. Mice were stabilized for 5 minutes. Then the increasing doses of methacholine (MCH) aerosol were administered for 20 seconds in increasing concentrations from 3.125 mg/ml to 50 mg/ml. The values of airway resistance were recorded by the system after the administration of MCH. After each dose, the data were continuously collected for 3 min and maximum values of lung resistance were taken to express changes in murine airway function.

### Analysis of bronchoalveolar lavage fluid (BALF) and serum

After the airway physiology analysis, blood was taken from the hearts of the mice. Then serum samples were collected after centrifugation (3000×g, 15 min, 4°C) and stored at −80°C until analyses. The airway lumina of each mouse were washed with three successive 0.5 mL volumes of PBS. The bronchoalveolar lavage fluid (BALF) was centrifuged and the upper fluid sample was retained for IL-4, IL-13 and TGF-β1 detection. The cell pellets were suspended for the cell counts. Total cells were counted with a haemocytometer. Differential cell counts were conducted using Wright's staining by counting at least 200 cells.

The IL-4, IL-13, TGF-β1 and OVA-specific IgE levels were measured with the method of enzyme linked immunosorbent assay (ELISA) according to manufacturer protocol. Concentrations were determined in duplicate for each sample. The OVA specific IgE ELISA kit was purchased from Biolegend (San Diego, USA). The ELISA kits of IL-4, IL-13 and TGF-β1 were purchased from Jingmei Company (Shenzhen, China).

### Lung histology analysis

After BALF samples were obtained, left lungs were fixed in formalin, embedded in paraffin, sectioned at 4 μm thickness, and stained with hematoxylin-eosin (H&E) solution and periodic acid schiff (PAS) to estimate inflammation and mucus production, respectively. Histological analyses were performed by pathologists blinded to the four groups. The numbers of PAS-negative and PAS-positive epithelial cells (goblet cells) in individual bronchioles were counted. The percentage of PAS-positive cells per bronchiole was calculated based on the number of PAS positive epithelial cells divided by the total number of epithelial cells. At least ten bronchioles were counted in each slide randomly.

Immunohistochemistry was employed to identify α-smooth muscle actin (SMA). Sections were deparaffinized and rehydrated in graduated alcohol solutions. Endogenous peroxidase activity was blocked by 3% H2O2. After 5% normal goat serum, specimens were incubated with mouse anti-human α-SMA moloclonal antibody (DAKO, Glostrup, Denmark) at dilution of 1∶50. Substitution of primary antibody with normal rabbit IgG was used as a negative control. The slides were incubated overnight and rinsed with PBS, and then incubated with peroxidase-labeled goat anti-mouse polyclonal IgG (1∶1000 dilution; DAKO, Glostrup, Denmark) for 30 min at 37°C, and then washed again with PBS followed diaminobenzidine (Santa Cruz Biotechnology, Santa Cruz, USA) stain. The sections were counterstained with hematoxylin. Immunostained sections were analyzed using Image-Pro Plus 6.0 software (Media Cybernetics, Bethesda, MD, USA). Data were expressed as the area of α-SMA immunostaining per micrometer length of basement membrane of bronchioles with 150−200 μm internal diameter. At least 10 bronchioles in each slide were randomly selected to identify α-SMA.

Masson’s Trichrome staining was performed to evaluate subepithelial deposition of collagen. Positive trichrome-stained areas were outlined and quantified using Image-Pro Plus 6.0. Results are shown as the area of trichrome staining per micron length of basement membrane of bronchioles 150−200 μm of internal diameter. At least 10 bronchioles were randomly selected in each of the slides.

Total lung collagen content was determined according to the protocol of a commercial hydroxyproline detection kit (Nanjing Jiancheng Biotechnology Company, China).

### Western blot analysis of α-SMA and MMP-9 in lung tissue

Lung tissues were added to tissue lysis/extraction Extraction reagent plus protease inhibitor (Sigma-Aldrich) and homogenized. After absolute schizolysis and centrifugation at 12,000 × g for 15 min at 4°C, the supernatants were collected as the total protein samples. The protein concentrations were determined using the method of Bradford. The proteins (40 μg) were separated by 12% sodium dodecyl sulfate-polyacrylamide gel electrophoresis (SDS-PAGE) and transferred onto a polyvinylidene difluoride membrane. The membrane was incubated in blocking solution (5% dried milk in Tris-buffered saline) for 1 h at room temperature and then exposed to mouse anti-human α-SMA moloclonal antibody (1∶1000 dilution; DAKO, Glostrup, Denmark), mouse anti-human MMP-9 monoclonal antibody (1∶1000 dilution; Santa Cruz Biotechnology, Santa Cruz, CA) and mouse anti-human GAPDH monoclonal antibody (1∶1000 dilution; Cell Signaling Technology, Danvers, MA) overnight at 4°C with gentle shaking. Then the membrane was incubated with peroxidase-conjugated goat anti-mouse polyclonal IgG (1∶1000 dilution; DAKO, Glostrup, Denmark) for 1 h at 37°C and visualized with diaminobenzidine. Signals were quantified using the Fuji Image Gauge software program (ver. 3.0, Fuji Film, Tokyo, Japan).

### Statistical analysis

Data were presented as means±S.E.M. Statistical comparisons were made with Mann-Whitney test using SPSS 16.0. *P* < 0.05 was considered statistically significant.

## Results

### Effect of T0901317 on ABCA-1 mRNA in lung

**Figure 1:**
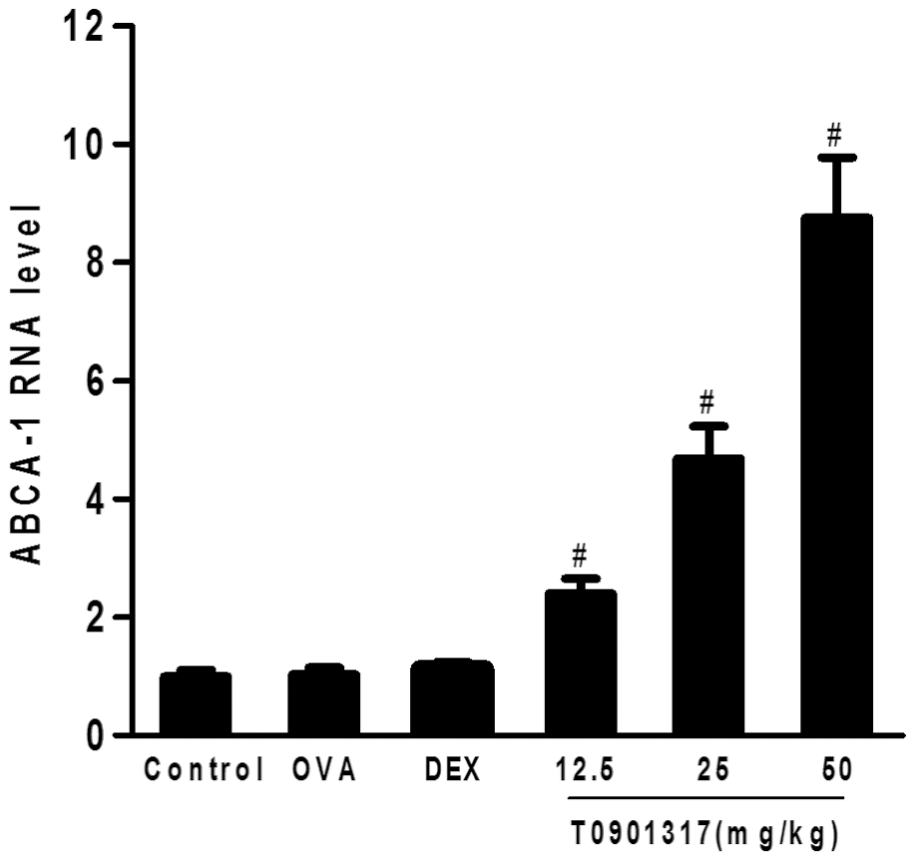
Treatment with T0901317 reduces ABCA-1 mRNA expression in the lungs. Ag-sensitized famale BAL/b C mice were treated with DEX or T0901317 before challenge, and samples were taken 24 h after the first challenge. ABCA-1 mRNA gene expression was measured in the lung tissue using real-time PCR and expressed as fold difference from control mice. Three independent experiments were examined (5 mice in each group of one experiment). * Significant differences (*P*<0.05) between the control group and the OVA group, # Significant differences (*P*<0.05) between the OVA group and T0901317 group.

Whole-lung mRNA expression was determined using quantitative RNA analysis. Treatment with T0901317 caused a dose-related, statistically significant increase in ABCA-1 mRNA expression in the lung tissue (Fig. 1). This indicated that the ligand is actually activating the LXR in this model system, suggesting that the dosing regimen used is appropriate and that this ligand is effective in mice.

### Effect of T0901317 on airway inflammation in chronic model of asthma

**Figure 2:**
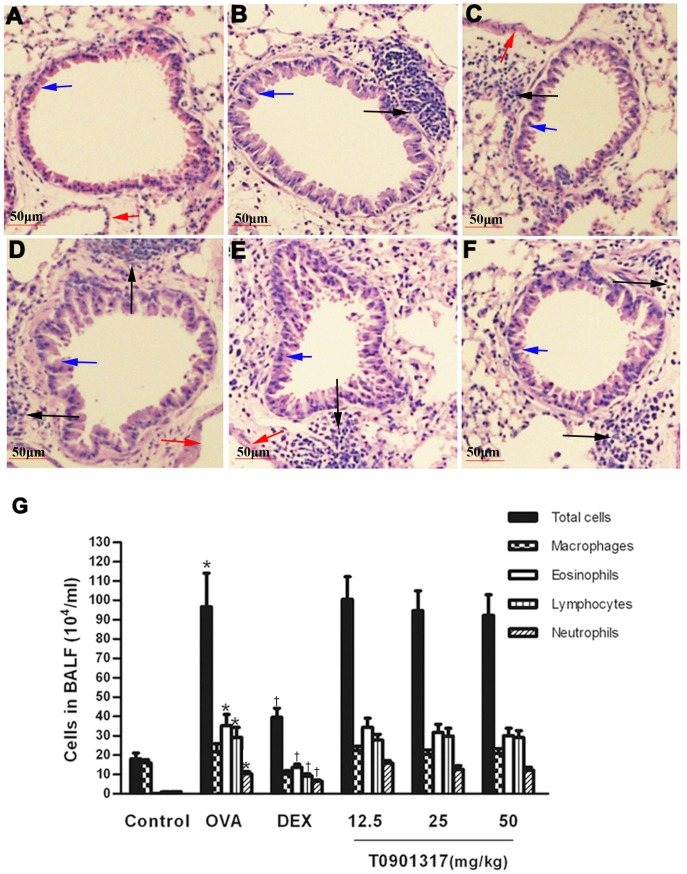
LXR ligand does not affect allergic airway inflammation and inflammatory cells in BALF. Examination of lung tissue was performed after the last OVA challenge. Lung tissues were fixed, sectioned at 4 mm thickness, and stained with H&E solution (magnification×200). The inflammatory cells (black arrows) around the bronchial epithelial cells (blue arrows) and vessels (red arrows) were observed by pathologists blinded to the four groups. (A) Control group, (B) OVA group, (C) DEX group and (D-F) T0901317 group (12.5, 25, 50 mg/kg bodyweight). Cells were isolated by centrifugation and stained with Wright's stain reagent. Cell numbers and cell differentiation (G) in BALF were determined using a hemocytometer to count at least 200 cells. Three independent experiments were examined (6 mice in each group of one experiment), * Significant differences (*P*<0.05) between the control group and the OVA group, † Significant differences (*P*<0.05) between the OVA group and DEX group, # Significant differences (*P*<0.05) between the OVA group and T0901317 group.

The inflammation of airway and the amount of inflammatory cells in the BALF were determined. Few inflammatory cells were found in the lungs of control group. Mice in OVA group displayed severe infiltration of inflammatory cells around the respiratory tract and vessels. Mice treated with DEX showed marked reduction of the inflammatory cells around the airway. However, T0901317 failed to reduce the OVA-induced inflammation (Figure. 2A-F). Following sensitization and challenges, numbers of total leukocytes, as well as numbers of lymphocytes, neutrophils and eosinophils in BALF were significantly increased compared to control group. Treatment with DEX significantly reduced numbers of all cell types in the BALF. However we didn’t find the depression of inflammatory cells in BALF in the mice treated with T0901317 (Figure. 2G).

### Effects of T0901317 on production of Th2-cytokines and OVA-specific IgE

**Figure 3:**
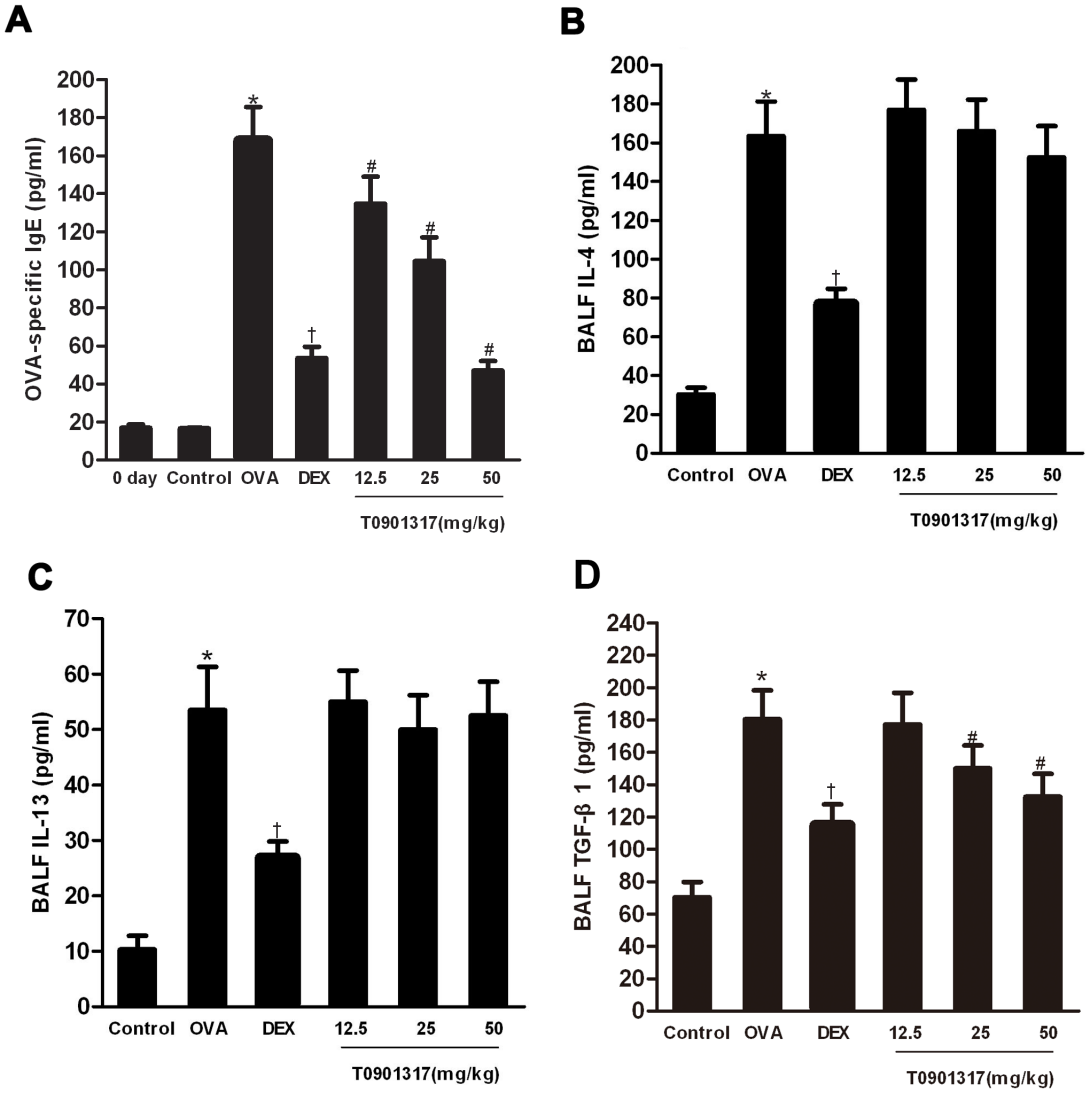
LXR ligand reduces TGF-β1 in BALF and serum OVA-specific IgE in chronic asthmatic process but has no effect on IL-4 or IL-13 in BALF. Serum OVA-specific IgE (A) and cytokine levels of IL-4 (B), IL-13 (C) and TGF-β1 (D) in BALF were measured by ELISA. Three independent experiments were examined (6 mice in each group of one experiment). * Significant differences (*P*<0.05) between the control group and the OVA group, † Significant differences (*P*<0.05) between the OVA group and DEX group, # Significant differences (*P*<0.05) between the OVA group and T0901317 group.

Allergic asthma is characterized by overproduction of IL-4, IL-13 and high level of serum IgE. Following sensitization and challenges, IL-4 and IL-13 in BALF and serum OVA-specific IgE were markedly increased compared with those of the control group. The administration of T0901317 significantly reduced the levels of serum OVA-specific IgE but not Th2 cytokines relative to those in the OVA group while DEX treatment reduced both IgE and Th2 cytokines in the OVA-challenged mice (Figure. 3A-C).

### Effect of T0901317 on AHR in chronic experimental asthma

**Figure 4:**
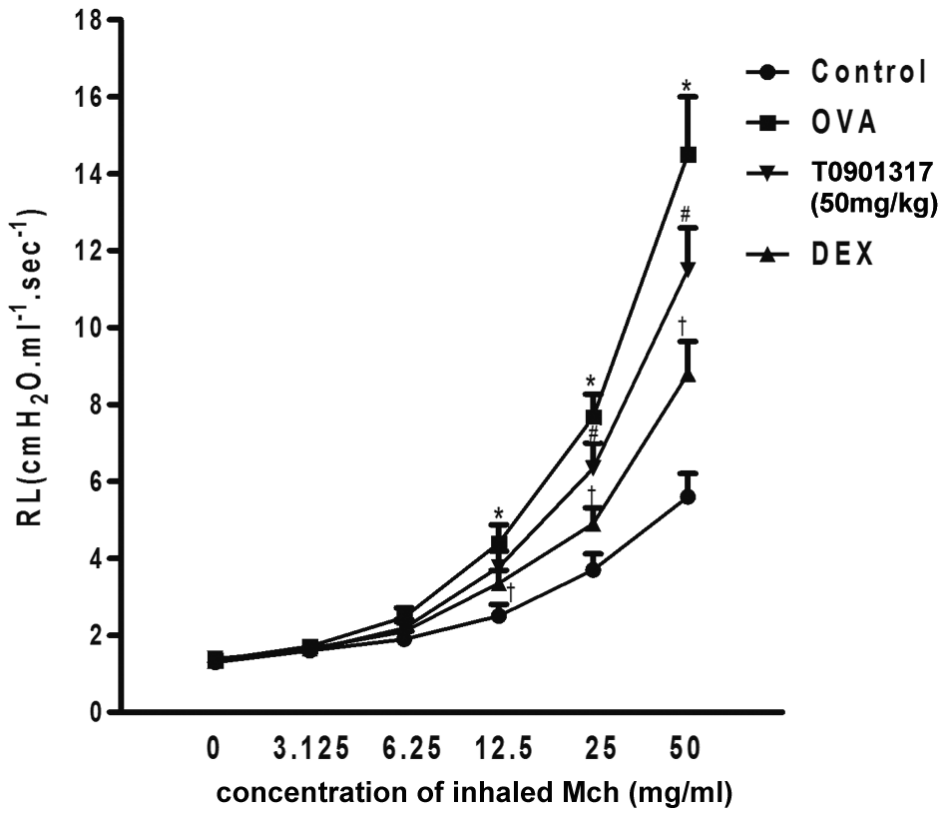
Treatment with T0901317 reduces allergen-induced airway hyperresponsiveness in response to MCH. Changes in lung resistance were recorded for 3 min after challenge with aerosolized MCH for 20 sec at the indicated doses 24h after OVA challenge. Mice in OVA group received 3 intraperitoneal injections of OVA emulsified with aluminum hydroxide on days 0, 7, and 14. They were then challenged with aerosolized OVA 3 days/ week for 8 weeks. The control mice received sham sensitization and challenge. Mice of DEX group received intraperitoneal injections of DEX (1 mg/kg) 2 hours before every OVA challenge. Mice of T0901317 group received T0901317 (50 mg/kg bodyweight) orally 2 hours before OVA challenge.* Significant differences (*P*<0.05) between the control group and the OVA group, † Significant differences (*P*<0.05) between the OVA group and DEX group, # Significant differences (*P*<0.05) between the OVA group and T0901317 group.

In vivo airway reactivity to aerosolized MCH was assessed. We found that aerosolized MCH caused a dose-dependent increase in RL in all groups. At low doses of MCH (3.125 mg/mL), no statistical difference was noted in RL among the four groups. At doses of MCH ≥12.5 mg/mL, RL in the DEX group was significantly lower than that of the control group. At doses of MCH ≥25 mg/mL, RL was markedly lower in the T0901317 group (50 mg/kg) than that of the OVA group (Figure. 4). But we didn’t find the depression of RL in the mice treated with low and moderate dose of T0901317 (data not show).

### T0901317 inhibits airway remodeling in chronic experimental asthma

**Figure 5:**
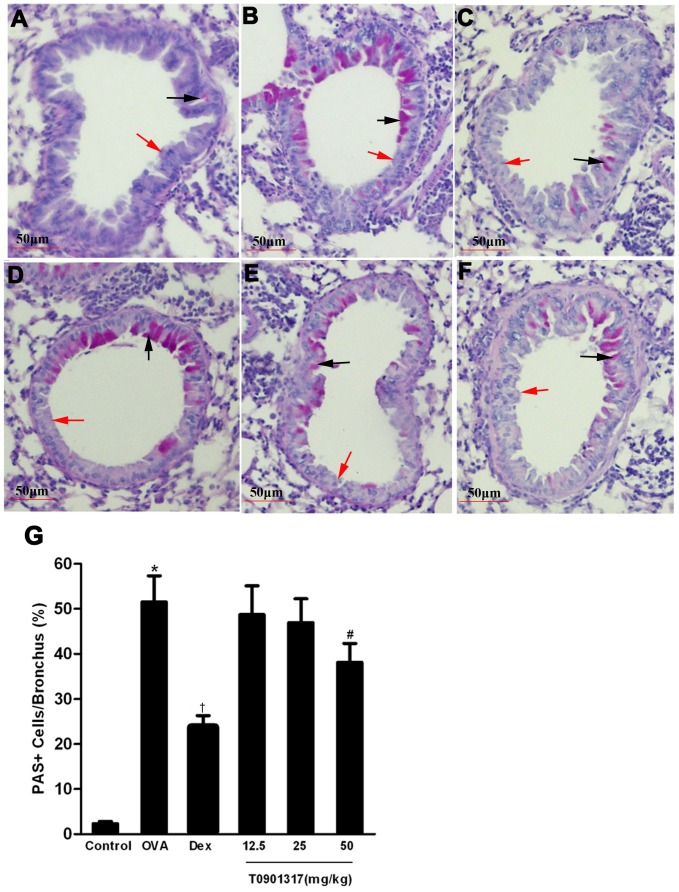
Treatment with T0901317 reduces OVA-induced mucus production. Lung tissues were fixed, sectioned at 4 μm thickness, and stained with Periodic Acid Schiff for mucus production (magnification ×200). The PAS-positive epithelial cells (black arrows) and PAS-negative epithelial cells (red arrows) were counted. (A) Control group, (B) OVA group, (C) DEX group and (D-F) T0901317 group (12.5, 25, 50 mg/kg bodyweight). The percentage of PAS-positive cells per bronchiole (G) was calculated. Quantitative analysis was assessed in at least ten bronchioles of a sample slide stained with PAS. Results are expressed as mean ± SEM (6 mice in each group of one experiment) of three independent experiments.* Significant differences (*P*<0.05) between the control group and the OVA group, † Significant differences (*P*<0.05) between the OVA group and DEX group, # Significant differences (*P*<0.05) between the OVA group and T0901317 group.

The level of airway metaplasia and mucus production was assessed by PAS staining of lung tissues and the percentage of PAS positive cells of total epithelial cells was determined. Overproduction of mucus and goblet cell hyperplasia were observed in bronchial airways of mice in OVA group. In contrast, DEX treatment mice showed a reduction in the number of PAS-stained goblet cells. Large dose T0901317-treated animals displayed a significant reduction in numbers of goblet cells in the airway epithelium to about 24% as compared with control mice (Figure. 5).

**Figure 6:**
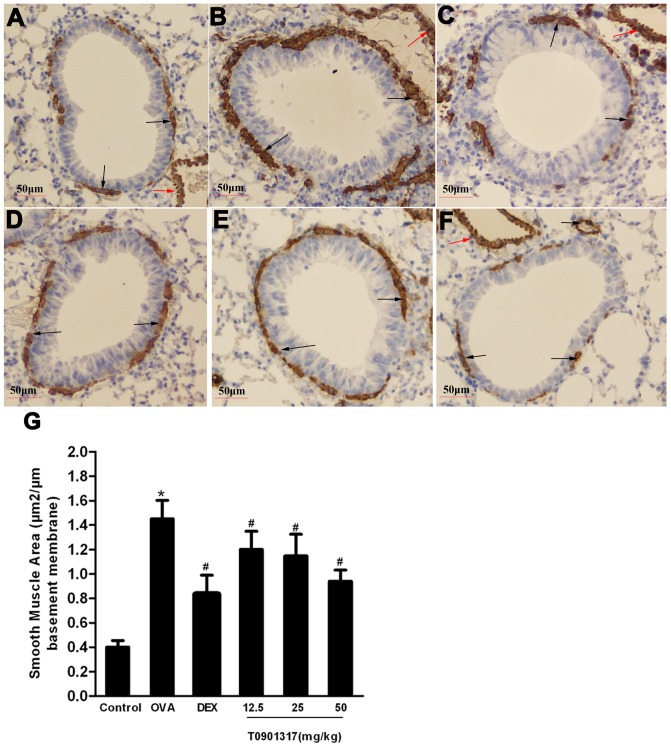
LXR ligand inhibits the areas of α-SMA in murine model of asthma. Immunohistochemistry was employed to identify α-SMA (magnification×200). The immunostained areas around bronchioles (black arrows) were analyzed using Image-Pro Plus 6.0 software. The red arrows indicate the α-SMA immunostained areas of vessels. (A) Control group, (B) OVA group, (C) DEX group and (D-F) T0901317 group (12.5, 25, 50 mg/kg bodyweight). The area of α-SMA per micrometer length of basement membrane of bronchiole (G) was calculated. Three independent experiments were examined (6 mice in each group of one experiment). * Significant differences (*P*<0.05) between the control group and the OVA group, † Significant differences (*P*<0.05) between the OVA group and DEX group, # Significant differences (*P*<0.05) between the OVA group and T0901317 group.

**Figure 7:**
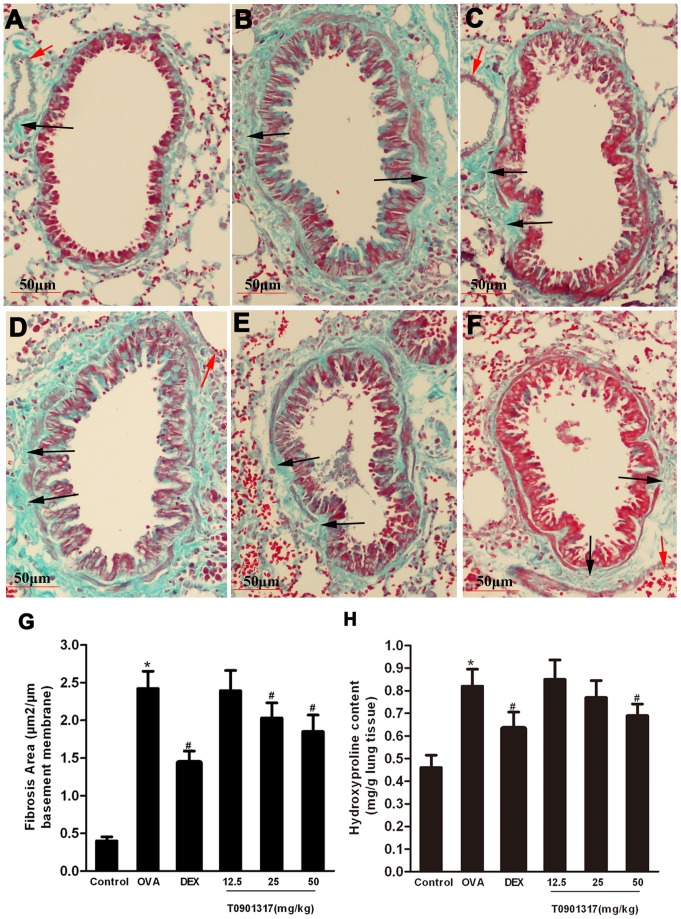
LXR ligand inhibits pulmonary α-SMA and MMP-9 protein accumulation in chronic experimental asthma. (A) Total proteins of lung tissue were extracted 24 h after the final OVA challenge and subjected to Western blot analysis of α-SMA and MMP-9. GAPDH was utilized as the standard control. (B, C) The band signal strength of α-SMA and MMP-9 expressed as a ratio to GAPDH. T0901317 inhibited α-SMA and MMP-9 expression in a dose-dependent manner in chronic model of asthma. Three independent experiments were examined (6 mice in each group of one experiment). * Significant differences (*P*<0.05) between the control group and the OVA group, † Significant differences (*P*<0.05) between the OVA group and DEX group, # Significant differences (*P*<0.05) between the OVA group and T0901317 group.

Additionally, α-SMA was increased in the peribronchiolar region of OVA challenged mice as compared to the control mice. Administration of T0901317 and DEX evidently decreased the areas of α-SMA-stained smooth muscle layer compared with the OVA group (Figure. 6). Western blot analysis of whole lung lysates also showed significant increase of α-SMA in the mice of OVA group compared with the control group. This increase was attenuated in mice treated with DEX. Furthermore, we also observed T0901317 reduced the α-SMA protein in lung in a dose-dependent manner (Figure. 7). So it can be clearly seen that the LXR ligand reduces the thickness of airway smooth muscle (ASM).

**Figure 8:**
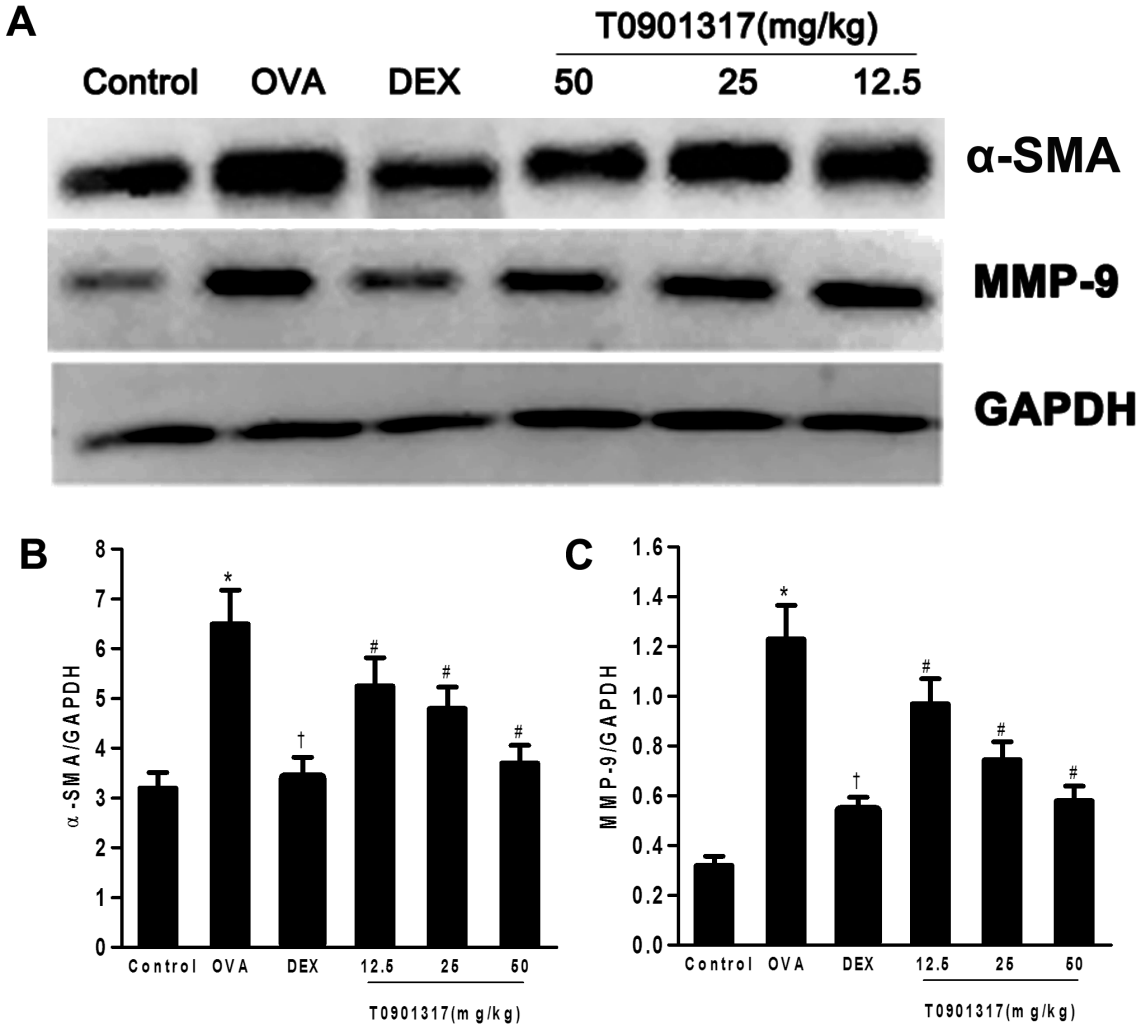
LXR ligand inhibits peribronchial fibrosis in chronic experimental asthma. Masson’s Trichrome staining was performed to evaluate subepithelial deposition of collagen. Positive trichrome-stained areas (black arrows) around the bronchioles and vessels (red arrows) were outlined and quantified using Image-Pro Plus 6.0. (A) Control group, (B) OVA group, (C) DEX group and (D-F) T0901317 group (12.5, 25, 50 mg/kg bodyweight). The area of trichrome staining per micron length of basement membrane of bronchiole (G) was calculated. At least 10 bronchioles were randomly selected in each of the slides. (H) Total lung hydroxyproline content was determined according to the protocol of a commercial hydroxyproline detection kit. Three independent experiments were examined (6 mice in each group of one experiment). * Significant differences (*P*<0.05) between the control group and the OVA group, † Significant differences (*P*<0.05) between the OVA group and DEX group, # Significant differences (*P*<0.05) between the OVA group and T0901317 group.

Masson’s trichrome staining revealed dense collagen deposition/fibrosis throughout the interstitium in airways and vessels of tissues from the mice in OVA group. The mean area of airway fibrosis in the OVA group was profoundly enhanced compared with the negative control group. Administration of DEX caused reduction of the collagen deposition compared with the OVA group. We also observed T0901317-treated animals displayed a significant reduction of fibrosis area in a dose-dependent manner compared with the OVA group (Figure. 8A-G).

Total lung hydroxyproline content in the OVA group was significantly greater than that in the control group. In contrast, treatment with T0901317 (50 mg/kg) and DEX obviously reduced total lung hydroxyproline content compared with the OVA group (Figure. 8H). But we did not find the depression of hydroxyproline content in the mice treated with low and moderate dose of T0901317. These results indicate that LXR acts to control allergen induced airway fibrosis in the lung.

### T0901317 inhibits MMP-9 and TGF-β1 in chronic model of asthma

To clarify the mechanism of LXRs in remodeling process of asthma, we investigated the TGF-β1 in BALF and MMP-9 expression in lungs of animals. ELISA analysis showed a significant increase of TGF-β1 in the BALF of OVA-challenged mice. The mice treated with DEX and T0901317 showed lower level of TGF-β1 in BALF compared with the OVA group (Fig. 3D).

As shown in Fig. 7, OVA-challenged mice showed marked expression of MMP-9 compared with the normal controls. The increase was reduced by the DEX treatment. We also observed T0901317 treatment reduced MMP-9 in a dose-dependent manner in lung tissue of OVA-challenged mice.

## Discussion

Asthma is one of the most common chronic diseases worldwide. It is considered an allergic disease with characteristic airway inflammation and airway remodeling.

In this study we evaluate the effect of LXRs on airway remodeling using a long-term exposure murine model of asthma. In our murine model of asthma we were able to demonstrate that T0901317 activated LXRs, suggesting that the dosing regimen used was appropriate.

A key feature of bronchial asthma is the high expression of IgE. Allergic asthma is mediated by the triggering of IgE-bound mast cells by allergens leading to the release of vasoactive mediators. As the key effector molecule of type I hypersensitivity, IgE is critical for allergic immune reaction and AHR [17]. Previous studies have demonstrated that Liver X receptors attenuate IgE expression in B cells [18] and high-affinity IgE receptor-stimulated mast cell activation [19]. In our study, OVA-specific IgE production was blocked by T0901317 in animal models of asthma. This effect may partially account for the suppression of AHR.

The inflammation of microenvironment present within the lung is fundamental for asthma. IL-4 and IL-13 have an important role in Th2-mediated inflammatory events, which have been demonstrated in IL-4-deficient and IL-13-deficient mice [20], [21]. In our study, sensitization and chronic challenge with OVA resulted in significant inflammation in airway of mice, including recruitment of inflammatory cells and production of Th2 cytokines. T0901317 failed to reduce OVA-induced Th2 cytokines and inflammatory cells in lung. Therefore LXR agonist was not as effective as glucocorticoid in inhibiting the Ag-induced chronic inflammation.

The airway remodeling denotes the pathophysiologic modifications of airway wall structure, including epithelial denudation, subepithelial fibrosis, mucus gland hypertrophy, myofibroblast and smooth muscle proliferation, and angiogenesis [20], [21]. Airway remodeling is the major cause of symptoms associated with decreased pulmonary function. We demonstrated for the first time that T0901317 significantly suppresses the deposition of subepithelial collagen and the hyperplasia of ASM in chronic experimental asthma. Large dose of T0901317 reduced the number of mucus producing goblet cells within the epithelium after OVA challenge. So LXR agonist has inhibitory effect on airway remodeling. As T0901317 did not abolish the chronic airway inflammation, we explored other likely reasons that could contribute to attenuate airway remodeling.

There are several mediators of remodeling have been identified, including TGF-β1 and MMP-9. Our study showed LXR agonist reduced the high levels of TGF-β1 and MMP-9 in the murine model of asthma. The pro-fibrotic cytokine TGF-β1 was suggested to induce proliferation of fibroblast cells and their differentiation into myofibroblasts and extracellular matrix protein synthesis during the development of subepithelial fibrosis [22]. Levels of TGF-β1 are significantly increased in patients with severe asthma [23], [24]. So we guess the ability of LXRs to attenuate the expression of TGF-β1 may partially lead to the suppression of remodeling.

MMP-9 is one of the proteases with abilities to cleave components of the extracellular matrix (ECM). It plays an important role in the tissue remodeling associated with various pathological processes such as morphogenesis, angiogenesis, tissue repair, migration and metastasis [25], [26]. Previous study has shown that liver X receptors inhibit the expression of cytokine-induced MMP-9 in macrophages [5]. So we guess the LXRs-mediated reduction of MMP-9 may partially account for the anti-fibrosis activity of LXRs.

T0901317 is one of the synthetic ligands of LXR. Another ligand of LXR, GW 3965 has been shown the ability to increase AHR in acute allergic asthma [27]. The reason for this difference is not known. Mainly T0901317 could activate other nuclear receptors such as Farnesoid x receptor (FXR) [28]. The activation of FXR can induce vascular smooth muscle cell death and suppress smooth muscle cell migration [29]. This might be the putative mechanism of T0901317 in airway remodeling in chronic asthma.

The ability of T0901317 to limit remodeling makes it an attractive target for the treatment of chronic airway diseases. However, systemic use of T0901317 failed to significantly affect Ag-induced inflammatory mediator production or airway inflammation. As we all know, corticosteroid is the most effective treatment for controlling allergic inflammation. But the ability of corticosteroid to prevent airway remodeling is controversial [30]. So T0901317 could be potentially used in combination with glucocorticoids in asthma treatment.

In conclusion, we have shown that treatment with LXR ligand effectively inhibited allergen-induced airway remodeling. The ability of LXR ligand to modulate chronic asthma pathology makes it a promising target for asthma therapy.
